# Gallic Acid Enhances the Anticancer Activity of Docetaxel in Triple-Negative Breast Cancer Cells

**DOI:** 10.3390/biology15141131

**Published:** 2026-07-11

**Authors:** Mehmet Emin Ayağ, Mehmet Cudi Tuncer, İlhan Özdemir

**Affiliations:** 1 Mehmet Emin Ayağ Practice, Department of Gynecology and Obstetrics, 47000 Mardin, Turkey; 2 Department of Anatomy, Faculty of Medicine, Dicle University, 21280 Diyarbakır, Turkey; drcudi@hotmail.com; 3 Department of Histology and Embryology, Faculty of Medicine, Kahramanmaraş Sütçü İmam University, 46000 Kahramanmaraş, Turkey; ilhanozdemir32@hotmail.com

**Keywords:** gallic acid, docetaxel, MDA-MB-231, HaCaT, apoptosis, cell viability

## Abstract

Experimental evidence has shown that gallic acid (GA), a naturally occurring polyphenolic compound, and docetaxel (DTX), a taxane chemotherapeutic agent, each possess antitumor activity against multiple cancer types. Although both compounds have been investigated individually, their combined effects in triple-negative breast cancer (TNBC) have received limited attention, and the molecular basis of their interaction remains unclear. The present study examined the in vitro effects of GA and DTX in MDA-MB-231 TNBC cells while simultaneously assessing their comparative cytotoxicity in HaCaT human keratinocytes. Evaluation of treatment efficacy included measurement of cell viability by the MTT assay and assessment of drug interactions using the Chou–Talalay combination index (CI) method. Apoptosis together with cell-cycle distribution was subsequently examined using both Annexin V/PI flow cytometry and TALI^®^ image-based cytometry. Additional analyses included β-tubulin immunofluorescence (IF), caspase-9 immunocytochemistry, ELISA, wound-healing assays, quantitative real-time PCR, and bioinformatic analyses to investigate treatment-associated biological alterations. Combined exposure to GA and DTX produced a significant reduction in cell viability and exhibited synergistic activity in MDA-MB-231 cells. The coordinated biological response to the combined treatment was characterized by increased apoptotic cell death, arrest of the cell cycle at the G2/M phase, extensive disorganization of the β-tubulin network, and enhanced caspase-9 immunoreactivity. Beyond its effects on cell survival, the combined regimen substantially decreased the release of IL-6, IL-8, and TNF-α, limited wound-healing capacity, and reshaped the expression profile of the apoptosis- and cell cycle-related genes *BCL2*, *BAX*, *CASP9*, and *CDKN1A*. Bioinformatic analyses further revealed enrichment of apoptosis- and cell-cycle-associated pathways that were generally consistent with the experimental observations. The overall pattern of experimental responses indicates that combining GA with DTX enhances the in vitro antitumor efficacy of DTX in TNBC cells by simultaneously influencing apoptotic pathways, cell-cycle regulation, inflammatory cytokine secretion, and cellular migratory capacity. Although the bioinformatic findings provide supportive hypothesis-generating evidence, additional studies using three-dimensional models, in vivo experiments, and functional validation approaches are necessary to confirm the underlying molecular mechanisms and to further define the translational potential of this therapeutic combination.

## Simple Summary

Limited therapeutic options and the rapid emergence of chemoresistance continue to make triple-negative breast cancer one of the most challenging breast cancer subtypes to manage effectively. Consequently, considerable interest has focused on combining naturally derived bioactive compounds with established chemotherapeutic agents to enhance antitumor efficacy while potentially permitting dose optimization of conventional drugs. In the present study, the ability of gallic acid to augment the biological activity of docetaxel was investigated using a triple-negative breast cancer cell model. Compared with either monotherapy, the combined treatment consistently produced stronger antitumor effects, including a greater reduction in cell viability, enhanced apoptotic cell death, increased G2/M cell-cycle arrest, suppression of pro-inflammatory cytokine secretion, and impaired migratory capacity. These experimental findings were further supported by gene expression analyses demonstrating upregulation of pro-apoptotic signaling together with downregulation of anti-apoptotic pathways. Complementary network pharmacology analyses also identified several biologically relevant signaling pathways that may participate in the observed responses, although these computational findings require further experimental validation. Collectively, the present results demonstrate that gallic acid enhances the in vitro antitumor activity of docetaxel against triple-negative breast cancer cells. Nevertheless, confirmation of these findings in three-dimensional culture systems, well-designed preclinical animal models, and ultimately clinical investigations will be necessary before the therapeutic relevance of this combination can be fully established.

## 1. Introduction

Worldwide, breast cancer represents the most prevalent malignancy among women and is responsible for a substantial proportion of cancer-related deaths [1]. Triple-negative breast cancer (TNBC) is distinguished by the absence of estrogen receptor (ER), progesterone receptor (PR), and human epidermal growth factor receptor 2 (HER2), a molecular profile that is associated with aggressive tumor biology, restricted treatment opportunities, and unfavorable clinical outcomes [1,2]. Owing to the limited availability of effective targeted therapies, systemic chemotherapy remains the principal treatment strategy for TNBC. Nevertheless, the clinical benefits of chemotherapy are often diminished by treatment-related toxicity, the emergence of drug resistance, and other adverse effects that restrict therapeutic efficacy [3].

As a taxane-based chemotherapeutic agent, docetaxel (DTX) has become an established component of treatment strategies for several solid malignancies, including breast, non-small cell lung, prostate, gastric, and head and neck cancers [4]. By promoting microtubule stabilization and preventing their normal dynamic reorganization, DTX interrupts mitotic progression, induces G2/M-phase arrest, and subsequently initiates apoptosis in susceptible tumor cells [5]. Although DTX has demonstrated substantial clinical effectiveness, its therapeutic application is frequently complicated by toxicities such as myelosuppression, peripheral neuropathy, and febrile neutropenia, often requiring dose reduction or premature discontinuation of treatment [6].

Gallic acid (GA; 3,4,5-trihydroxybenzoic acid) is a bioactive polyphenol widely distributed in edible plants and traditional medicinal species such as pomegranate, grapes, green tea, and oak bark [7]. Extensive experimental evidence indicates that this compound exhibits multiple pharmacological activities, including antioxidant, anti-inflammatory, antimicrobial, antidiabetic, and anticancer properties [8,9,10]. Rather than acting through a single mechanism, GA affects a broad range of molecular pathways involved in tumor progression. These include intrinsic and extrinsic apoptosis, regulation of reactive oxygen species (ROS) homeostasis, angiogenesis, and several cancer-related signaling pathways, including NF-κB, PI3K/AKT/mTOR, MAPK, and Wnt/β-catenin [11,12].

The combination of naturally occurring polyphenols with established chemotherapeutic drugs has emerged as a promising strategy to enhance anticancer efficacy while potentially minimizing treatment-associated toxicity [13,14,15]. Synergistic antitumor activity has been reported for several polyphenolic compounds, including rosmarinic acid, resveratrol, quercetin, and epigallocatechin-3-gallate (EGCG), when administered together with taxanes, platinum-based agents, or anthracyclines in experimental cancer models [16,17,18]. Although the anticancer potential of GA has been documented in a variety of malignancies, relatively little information is available regarding its combined use with DTX, particularly in models of TNBC [19].

Beyond the intrinsic characteristics of malignant cells, tumor progression is profoundly affected by the surrounding tumor microenvironment, which supplies inflammatory signals that facilitate cancer growth, invasion, metastasis, and resistance to treatment. Within this inflammatory network, interleukin-6 (IL-6), interleukin-8 (IL-8), and tumor necrosis factor-α (TNF-α) have emerged as major mediators of tumor-associated signaling pathways [20,21]. Many anticancer drugs eliminate tumor cells by triggering the intrinsic mitochondrial apoptotic pathway. This process involves mitochondrial outer membrane permeabilization (MOMP), release of cytochrome c, and sequential activation of caspases, ultimately leading to apoptotic cell death [22,23]. Since metastatic spread remains the major cause of cancer-related mortality, assessment of treatment-induced alterations in wound closure may provide additional insight into aggressive cellular behavior. The integration of computational network analysis with experimental research has expanded the ability to investigate treatment-associated molecular mechanisms. In particular, protein–protein interaction (PPI) network analysis together with Gene Ontology (GO) and Kyoto Encyclopedia of Genes and Genomes (KEGG) enrichment analyses enables the identification of candidate biological processes and signaling pathways that may contribute to therapeutic responses [24].

Although both GA and DTX have individually demonstrated anticancer activity, the biological consequences of their combined administration in TNBC have not been comprehensively investigated. On the basis of the available experimental evidence, we postulated that incorporating GA into DTX treatment would strengthen the antitumor response by concurrently modulating apoptosis, cell-cycle progression, inflammatory cytokine production, and cytoskeletal organization. Based on this hypothesis, the present study comprehensively examined the biological effects of GA and DTX, administered individually or in combination, in MDA-MB-231 TNBC cells while simultaneously evaluating comparative cytotoxicity in HaCaT human keratinocytes. To accomplish this objective, a multiparametric experimental approach incorporating cell viability analysis, apoptosis and cell-cycle evaluation, β-tubulin IF, caspase-9 immunocytochemistry, cytokine profiling, wound-healing assays, quantitative real-time PCR, and bioinformatic analyses was employed to characterize the cellular and molecular responses associated with combination treatment.

## 2. Materials and Methods

### 2.1. Experimental Cell Models and Culture Conditions

The experimental study was performed using the human triple-negative breast cancer cell line MDA-MB-231 (American Type Culture Collection, ATCC, Manassas, VA, USA) together with the immortalized human keratinocyte cell line HaCaT (CLS No. 300493; Cell Lines Service, Eppelheim, Germany). Both cell lines were maintained under the same culture conditions throughout the study unless otherwise specified. Cells were grown in Dulbecco’s Modified Eagle Medium (DMEM; Gibco, Thermo Fisher Scientific, Waltham, MA, USA) supplemented with 10% fetal bovine serum (FBS; Gibco, Thermo Fisher Scientific) and 1% penicillin–streptomycin (Gibco, Thermo Fisher Scientific). Cell cultures were incubated at 37 °C in a humidified incubator containing 5% CO_2_. Fresh culture medium was supplied every 2–3 days, and cells were routinely passaged at approximately 70–80% confluence using 0.25% trypsin–EDTA (Gibco, Thermo Fisher Scientific). To minimize passage-dependent variability and maintain experimental reproducibility, only cells between passages 5 and 20 were included in all experiments.

### 2.2. Preparation of GA and DTX Treatment Solutions

Gallic acid (GA; purity ≥ 99%; Sigma-Aldrich, St. Louis, MO, USA) and docetaxel (DTX; Sigma-Aldrich, St. Louis, MO, USA) were dissolved in dimethyl sulfoxide (DMSO; Sigma-Aldrich) to prepare concentrated stock solutions before each series of experiments. A 100 mM stock solution of GA was subsequently diluted with complete culture medium to obtain final treatment concentrations of 5, 10, 25, 50, 100, and 200 μM. Similarly, a 10 mM DTX stock solution was prepared in DMSO and serially diluted with complete culture medium to yield final concentrations of 0.1, 1, 5, 10, 25, and 50 nM.

To eliminate potential solvent-related effects, the final concentration of DMSO was standardized across all experimental conditions and maintained below 0.1% (*v*/*v*). Preliminary control experiments confirmed that DMSO at this concentration did not influence cell viability. Therefore, vehicle control cultures were exposed to complete culture medium containing 0.1% (*v*/*v*) DMSO, corresponding to the solvent concentration present in all GA- and DTX-treated groups.

### 2.3. Evaluation of Cytotoxicity and Drug Interaction by the MTT Assay

Cell viability following exposure to GA, DTX, or their combination was determined using the MTT colorimetric assay. MDA-MB-231 and HaCaT cells were seeded into 96-well plates at a density of 5 × 10^3^ cells per well and maintained overnight to allow cell attachment before treatment. The cultures were then incubated for 48 h with increasing concentrations of GA (5–200 μM), DTX (0.1–50 nM), or the corresponding combination treatments.

Upon completion of the 48 h treatment period, cellular metabolic activity was assessed by adding 10 μL of MTT reagent (5 mg/mL prepared in PBS; Sigma-Aldrich) to each well containing 100 μL of culture medium. After incubation for an additional 4 h at 37 °C, the generated formazan crystals were dissolved in 100 μL of DMSO, and absorbance was recorded at 570 nm using a microplate reader. Cell viability was calculated relative to untreated control cells, which were defined as 100%.

Dose–response curves were constructed by nonlinear regression analysis using GraphPad Prism version 9.0 (GraphPad Software, San Diego, CA, USA), and IC_50_ values were subsequently calculated. The interaction between GA and DTX was further characterized according to the Chou–Talalay method with CompuSyn software (version 1.0). Combination index (CI) values were determined over different fractional effect (Fa) levels and classified as synergistic (CI < 1), additive (CI = 1), or antagonistic (CI > 1). Based on the calculated IC_50_ and CI values, all subsequent mechanistic experiments were carried out using GA at its IC_50_ concentration together with DTX at one-half of its IC_50_ concentration (0.5 × IC_50_).

### 2.4. Quantification of Treatment-Induced Apoptosis by Annexin V/PI Flow Cytometry

Apoptotic cell death following treatment was quantified by Annexin V/propidium iodide (PI) flow cytometry using the FITC Annexin V Apoptosis Detection Kit I (BD Biosciences, San Jose, CA, USA). MDA-MB-231 cells were treated for 48 h with GA (IC_50_), DTX (IC_50_), or the corresponding combination regimen. After the treatment period, cells were harvested by trypsinization, washed twice with ice-cold phosphate-buffered saline (PBS), and resuspended in 1× binding buffer to obtain a final cell density of 1 × 10^6^ cells/mL.

For each experimental condition, 1 × 10^5^ cells were incubated with 5 μL of FITC-conjugated Annexin V and 5 μL of PI for 15 min at room temperature in the absence of light. Immediately thereafter, fluorescence signals were acquired using a BD FACSCanto II flow cytometer (BD Biosciences). A minimum of 20,000 events was collected for each sample to ensure robust quantitative analysis.

Flow cytometry data were processed using FlowJo software (version 10.8.1; BD Biosciences, Ashland, OR, USA). Based on the Annexin V/PI staining profile, cells were classified as viable (Annexin V^−^/PI^−^), early apoptotic (Annexin V^+^/PI^−^), late apoptotic (Annexin V^+^/PI^+^), or necrotic (Annexin V^−^/PI^+^). The relative proportion of each population was calculated and subsequently used for statistical analyses.

### 2.5. Independent Validation of Apoptosis by TALI^®^ Image-Based Cytometry

To independently verify the apoptotic response observed by flow cytometry, apoptosis was additionally assessed using the Tali^®^ Image-Based Cytometer (Thermo Fisher Scientific, Waltham, MA, USA) in combination with the Tali^®^ Apoptosis Kit (Alexa Fluor^®^ 488 Annexin V/PI). MDA-MB-231 cells were exposed to GA, DTX, or the combined treatment for 48 h before being harvested and processed according to the manufacturer’s instructions.

For apoptosis staining, cell suspensions were incubated with Alexa Fluor^®^ 488-conjugated Annexin V for 15 min at room temperature while protected from light. Propidium iodide (PI) was then added, and the incubation was continued for an additional 5 min. The stained cell suspensions were subsequently loaded onto Tali^®^ Cellular Analysis Slides and analyzed immediately using the Tali^®^ Image-Based Cytometer.

A minimum of 500 cells was evaluated for each experimental group. Based on fluorescence intensity and staining characteristics, the Tali^®^ analysis software (Thermo Fisher Scientific, Waltham, MA, USA) automatically classified cells as viable, apoptotic, or necrotic. The relative percentages of each cell population were then calculated and used for subsequent statistical analyses.

### 2.6. IF Assessment of β-Tubulin Organization

MDA-MB-231 cells were cultured on sterile glass coverslips for 24 h to permit adequate cellular attachment before treatment with GA (IC_50_), DTX (IC_50_), or their combination. At the completion of the 48 h treatment period, cells were fixed with 4% paraformaldehyde for 15 min, followed by permeabilization using 0.1% Triton X-100. Non-specific antibody binding was minimized by incubating the specimens with 1% bovine serum albumin (BSA) for 30 min.

Immunostaining was carried out by incubating the coverslips overnight at 4 °C with an anti-β-tubulin primary antibody (Sigma-Aldrich, SAB4200715). After washing to remove unbound primary antibody, the appropriate fluorophore-conjugated secondary antibody was applied for 1 h at room temperature. Cell nuclei were subsequently counterstained with DAPI, and fluorescence images were acquired using identical microscope settings for all experimental groups to ensure direct comparison.

Representative immunofluorescence images were examined qualitatively to compare treatment-associated changes in β-tubulin distribution, microtubule organization, and overall cellular morphology among the different experimental groups.

### 2.7. Quantification of Treatment-Associated Cytokine Secretion by ELISA

Treatment-related changes in inflammatory cytokine secretion were evaluated by determining the concentrations of IL-6, IL-8/CXCL8, and TNF-α in conditioned media collected from MDA-MB-231 cells after 48 h of exposure to GA, DTX, or their combination. Upon completion of the treatment period, the culture supernatants were collected and centrifuged at 300× *g* for 5 min to eliminate residual cells and cellular debris. The resulting clarified supernatants were aliquoted and preserved at −80 °C until ELISA analysis.

Cytokine concentrations were quantified using commercially available human sandwich ELISA kits (Human IL-6, Human IL-8, and Human TNF-α ELISA Kits; R&D Systems, Minneapolis, MN, USA). Briefly, standards and experimental supernatants were dispensed into antibody-coated microplates and processed according to the manufacturer’s protocol through sequential incubation with the corresponding detection antibody and horseradish peroxidase (HRP)-conjugated detection reagent. Absorbance was measured at 450 nm using a microplate reader, and cytokine concentrations were calculated from the generated standard curves. To account for treatment-dependent differences in cell number, all cytokine values were normalized to the total cellular protein content determined by the Bradford assay and are presented as pg/μg protein.

### 2.8. Assessment of Wound Closure by the Scratch Assay

The effect of GA, DTX, and their combination on the wound-closing capacity of MDA-MB-231 cells was investigated using an in vitro scratch assay. Cells were seeded into 6-well plates at a density of 5 × 10^5^ cells per well and cultured until they reached approximately 90–95% confluence, forming a uniform monolayer. A straight scratch was then generated using a sterile 200 μL pipette tip (Axygen Scientific, Union City, CA, USA), after which the wells were carefully washed with PBS to remove detached cells and residual debris.

Fresh serum-free culture medium containing GA (IC_50_), DTX (IC_50_), or the combined treatment was subsequently added to the cells. Representative images of the wounded area were recorded immediately after scratch formation (0 h) and again after 48 h using an inverted phase-contrast microscope equipped with a digital imaging system. Quantitative analysis of wound closure was performed with the MRI Wound Healing Tool plugin in ImageJ software (version 1.54; National Institutes of Health, Bethesda, MD, USA) by comparing the remaining wound area after 48 h with the initial scratch area. The extent of wound closure was expressed as the percentage of wound recovery relative to the baseline (0 h) measurement.

### 2.9. Analysis of Treatment-Associated Gene Expression by Quantitative Real-Time PCR

Changes in gene expression induced by the different treatment regimens were analyzed in MDA-MB-231 cells using quantitative real-time PCR (qRT-PCR). Total RNA was extracted with TRIzol™ Reagent (Invitrogen, Thermo Fisher Scientific, Waltham, MA, USA) according to the manufacturer’s protocol. RNA concentration and purity were determined using a NanoDrop™ 2000 spectrophotometer (Thermo Fisher Scientific, Waltham, MA, USA). Subsequently, complementary DNA (cDNA) was generated from 1 μg of total RNA with the SuperScript™ IV VILO™ Master Mix (Thermo Fisher Scientific, Waltham, MA, USA).

Quantitative amplification was performed on a StepOnePlus™ Real-Time PCR System (Applied Biosystems, Thermo Fisher Scientific, Waltham, MA, USA) using SYBR™ Green Master Mix (Thermo Fisher Scientific, Waltham, MA, USA). Transcript levels of *BCL2*, *BAX*, *CASP9*, *IL6*, and *CDKN1A* were quantified, whereas *GAPDH* and *ACTB* served as the internal reference genes. Relative gene expression was determined using the 2^−ΔΔCt^ method after normalization to the geometric mean of the Ct values obtained for *GAPDH* and *ACTB*. The normalized expression values were expressed as fold changes relative to the untreated control group.

PCR amplification was initiated with an initial denaturation step at 95 °C for 10 min, followed by 40 amplification cycles consisting of 95 °C for 15 s, 60 °C for 30 s, and 72 °C for 30 s. To further assess the balance between pro-apoptotic and anti-apoptotic signaling, the *BAX*/*BCL2* expression ratio was calculated from the normalized relative expression values according to the following equation:

*BAX*/*BCL2* ratio = Relative expression of *BAX*/Relative expression of *BCL2*

Primer sequences used in this study are presented in Table 1.

### 2.10. Bioinformatic Identification of Candidate Molecular Targets and Functional Enrichment Analysis

To explore the molecular mechanisms underlying the combined biological effects of GA and DTX, a network pharmacology approach was employed. Potential targets associated with both compounds were retrieved from the SwissTargetPrediction (https://www.swisstargetprediction.ch/; accessed on 6 June 2026), PharmMapper (https://www.lilab-ecust.cn/pharmmapper/; accessed on 6 June 2026), and TCMSP (https://www.tcmsp-e.com/; accessed on 6 June 2026) databases. Genes associated with breast cancer and skin cancer were independently collected from GeneCards (https://www.genecards.org/; accessed on 6 June 2026), OMIM (https://www.omim.org/; accessed on 6 June 2026), and DisGeNET (https://www.disgenet.org/; accessed on 6 June 2026). Shared genes identified between the compound-target datasets and the disease-associated gene datasets were selected for subsequent bioinformatic analyses.

The shared target genes were imported into the STRING database (version 11.5; https://string-db.org/; accessed on 6 June 2026) to construct protein–protein interaction (PPI) networks using a minimum interaction confidence score of 0.7. The generated interaction networks were visualized and further analyzed with Cytoscape (version 3.9.1; National Resource for Network Biology, Seattle, WA, USA). Functional enrichment analyses were performed using the clusterProfiler package (version 4.0) implemented in R (version 4.2.0; R Foundation for Statistical Computing, Vienna, Austria). Gene Ontology (GO) enrichment analysis was conducted for the biological process (BP), molecular function (MF), and cellular component (CC) categories, together with Kyoto Encyclopedia of Genes and Genomes (KEGG) pathway analysis. GO terms and KEGG pathways satisfying both *p* < 0.05 and an FDR-adjusted q value < 0.05 were considered significantly enriched.

### 2.11. Statistical Analysis

All experiments were performed in three independent biological replicates (*n* = 3), and quantitative results are presented as mean ± standard deviation (SD). Statistical analyses were carried out using GraphPad Prism version 9.0 (GraphPad Software Inc., San Diego, CA, USA).

Differences among three or more experimental groups were evaluated by one-way analysis of variance (ANOVA) followed by Tukey’s multiple-comparisons post hoc test. Comparisons between two groups were assessed using the unpaired Student’s *t*-test. All statistical analyses were based on two-sided tests, and a *p* value < 0.05 was considered statistically significant. Throughout the figures, statistical significance is indicated as *p* < 0.05, *p* < 0.01, and *p* < 0.001.

Dose–response relationships and IC_50_ values were determined by nonlinear regression analysis in GraphPad Prism. Drug interactions between GA and DTX were assessed using the Chou–Talalay method implemented in CompuSyn software (version 1.0). Combination index (CI) values were interpreted as synergistic (CI < 1), additive (CI = 1), or antagonistic (CI > 1). In addition, Fa–CI plots and isobolograms were generated to further characterize the interaction profiles of the two compounds.

Relative gene expression determined by qRT-PCR was calculated using the 2^−ΔΔCt^ method after normalization to the geometric mean of the reference genes *GAPDH* and *ACTB*. ELISA and wound-healing datasets were analyzed by one-way ANOVA followed by Tukey’s post hoc test. Changes in β-tubulin organization and treatment-associated cellular morphology observed by immunofluorescence were interpreted qualitatively through comparison of representative staining patterns among the experimental groups. For the bioinformatic analyses, GO and KEGG enrichment results were considered statistically significant when both *p* < 0.05 and the FDR-adjusted q value were <0.05.

## 3. Results

### 3.1. Differential Cytotoxic Responses and Synergistic Interaction of GA and DTX in MDA-MB-231 and HaCaT Cells

**Figure 1:**
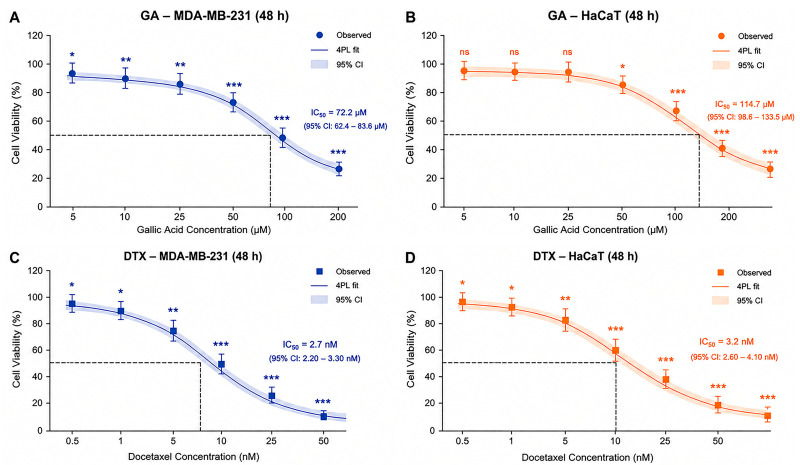
MTT-based evaluation of the cytotoxic activity of GA and DTX in MDA-MB-231 and HaCaT cells following 48 h of treatment. Nonlinear concentration-response curves were generated using a four-parameter logistic (4PL) regression model. Panels (**A**,**B**) present the viability profiles of MDA-MB-231 and HaCaT cells exposed to GA, with calculated IC_50_ values of 72.2 μM (95% CI: 62.4–83.6 μM) and 114.7 μM (95% CI: 98.6–133.5 μM), respectively. The corresponding responses to DTX are shown in panels (**C**,**D**), yielding IC_50_ values of 2.7 nM (95% CI: 2.20–3.30 nM) for MDA-MB-231 cells and 3.2 nM (95% CI: 2.60–4.10 nM) for HaCaT cells. Results are expressed as mean ± SD from three independent biological experiments (*n* = 3). Symbols indicate the experimental measurements, solid curves represent the fitted 4PL regression models, and the shaded areas correspond to the 95% confidence intervals. The dashed horizontal line denotes the 50% cell viability level, whereas the dashed vertical lines indicate the calculated IC_50_ values for each treatment. Statistical significance was assessed by one-way ANOVA followed by Tukey’s multiple-comparison test (* *p* < 0.05, ** *p* < 0.01, *** *p* < 0.001 versus the untreated control group, ns: not significant).

The cytotoxic profiles of GA and DTX were characterized in MDA-MB-231 and HaCaT cells after 48 h of exposure using the MTT assay. Analysis of the concentration-response curves demonstrated that increasing concentrations of both compounds progressively reduced cell viability in the two cell lines (Figure 1). However, the magnitude of the response differed between the malignant and non-malignant cells.

GA displayed greater cytotoxic potency in MDA-MB-231 cells than in HaCaT cells, yielding an IC_50_ value of 72.2 μM (95% CI: 62.4–83.6 μM) in the TNBC cell line compared with 114.7 μM (95% CI: 98.6–133.5 μM) in HaCaT cells. In contrast, DTX exerted marked antiproliferative activity at nanomolar concentrations in both experimental models. The calculated IC_50_ values were 2.7 nM (95% CI: 2.20–3.30 nM) for MDA-MB-231 cells and 3.2 nM (95% CI: 2.60–4.10 nM) for HaCaT cells, indicating a comparable level of sensitivity to DTX under the conditions employed in this study (Figure 1).

**Figure 2:**
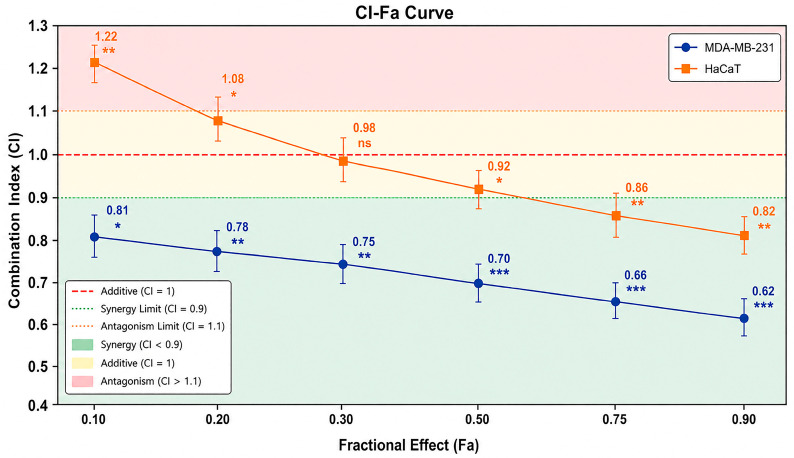
CI analysis describing the interaction profile of GA and DTX in MDA-MB-231 and HaCaT cells after 48 h of treatment according to the Chou–Talalay method. CI values were calculated over a range of Fa levels to characterize the pharmacological interaction between the two compounds. The blue curve represents the response of MDA-MB-231 cells, whereas the orange curve corresponds to HaCaT cells. The red dashed horizontal line indicates the theoretical additive interaction (CI = 1.0). The green and orange dotted lines define the limits for synergistic (CI = 0.9) and antagonistic (CI = 1.1) interactions, respectively. Shaded regions distinguish the three interaction categories: synergistic (CI < 0.9), additive (0.9 ≤ CI ≤ 1.1), and antagonistic (CI > 1.1). Data are presented as mean ± SD from three independent biological experiments (*n* = 3). Statistical significance was determined using a one-sample *t*-test by comparing the observed CI values with the theoretical additive value (CI = 1.0) (* *p* < 0.05, ** *p* < 0.01, *** *p* < 0.001; ns, not significant).

To further define the pharmacological interaction between GA and DTX, CI values were calculated across multiple Fa levels using the Chou–Talalay method (Figure 2). The interaction profile differed markedly between the two cell lines. In MDA-MB-231 cells, all calculated CI values remained below 0.90, ranging from 0.62 to 0.81, demonstrating a consistently synergistic interaction over the entire Fa range examined. Moreover, the magnitude of synergy became progressively greater as the fractional effect increased.

A different interaction pattern was observed in HaCaT cells. CI values varied between 0.82 and 1.22, indicating that the nature of the interaction depended on the treatment intensity. Antagonism predominated at lower Fa values, whereas an approximately additive response was obtained at Fa = 0.30 (CI = 0.98). At higher treatment effects (Fa ≥ 0.50), the interaction shifted from additivity toward synergism. Overall, these findings indicate that the combination of GA and DTX produced a more robust and reproducible synergistic effect in MDA-MB-231 cells than in the non-malignant HaCaT cell line.

Selection of the experimental treatment conditions for all subsequent mechanistic analyses was guided by the results of the cytotoxicity and CI studies. Based on these findings, MDA-MB-231 cells were exposed to GA at its IC_50_ concentration in combination with DTX at one-half of its IC_50_ value (0.5 × IC_50_). This concentration pair provided the basis for the remaining experiments and was used to investigate the cellular and molecular events associated with the synergistic interaction between the two compounds.

### 3.2. Combined Treatment with GA and DTX Enhances Apoptotic Cell Death in MDA-MB-231 Cells

**Figure 3:**
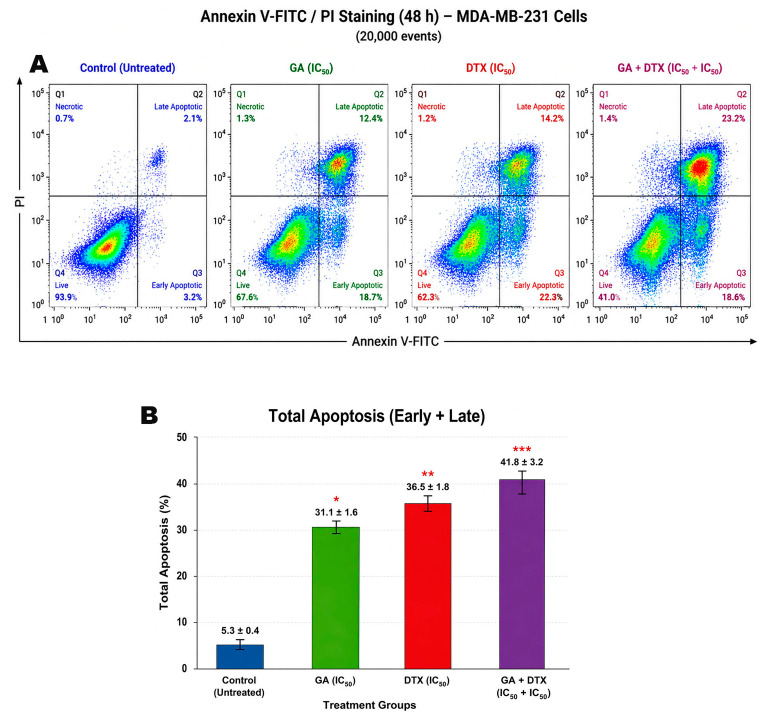
Flow cytometric analysis of apoptosis in MDA-MB-231 cells following 48 h of treatment with GA, DTX, or the combined regimen. (**A**) Annexin V-FITC/PI density plots illustrating the distribution of viable (Q4, Annexin V^−^/PI^−^), early apoptotic (Q3, Annexin V^+^/PI^−^), late apoptotic (Q2, Annexin V^+^/PI^+^), and necrotic (Q1, Annexin V^−^/PI^+^) cell populations. Values shown within each quadrant indicate the percentage of cells assigned to the corresponding population. A minimum of 20,000 events was acquired for each experimental condition. (**B**) Quantitative analysis of apoptosis expressed as the combined percentage of early and late apoptotic cells. Data are presented as mean ± SD from three independent biological experiments (*n* = 3). Statistical analysis was performed using one-way ANOVA followed by Tukey’s multiple-comparison test (* *p* < 0.05, ** *p* < 0.01, *** *p* < 0.001 versus the untreated control group).

The reduction in cell viability was further examined by determining whether it was accompanied by apoptotic cell death. For this purpose, MDA-MB-231 cells were stained with Annexin V-FITC/PI after 48 h of exposure to GA, DTX, or the combined treatment and analyzed by flow cytometry. The distribution of cell populations changed markedly following drug exposure, with a progressive loss of viable cells and a concomitant increase in apoptotic populations. Among all treatment groups, these alterations were most prominent after combined treatment (Figure 3A,B).

Flow cytometric analysis showed that untreated cultures consisted predominantly of viable cells (Q4, 93.9%), whereas early apoptotic (Q3, 3.2%), late apoptotic (Q2, 2.1%), and necrotic (Q1, 0.7%) populations remained low. Treatment with GA decreased the viable fraction to 67.6% while increasing the proportions of early and late apoptotic cells to 18.7% and 12.4%, respectively. A similar pattern was observed following DTX exposure, with viable cells accounting for 62.3% of the population and early and late apoptosis increasing to 22.3% and 14.2%, respectively. The combined GA + DTX regimen produced the largest redistribution of cell populations, reducing viability to 41.0% and increasing early and late apoptotic fractions to 18.6% and 23.2%, respectively. In contrast, the percentage of necrotic cells remained low in every experimental group, varying only between 0.7% and 1.4% (Figure 3A).

When early and late apoptotic populations were combined for quantitative analysis, a clear treatment-dependent increase in apoptosis became evident (Figure 3B). Total apoptosis rose from 5.3 ± 0.4% in untreated cells to 31.1 ± 1.6% following GA treatment (* *p* < 0.05) and to 36.5 ± 1.8% after DTX exposure (** *p* < 0.01). The highest apoptotic response was detected in the combination group, reaching 41.8 ± 3.2% (*** *p* < 0.001 versus untreated control). These quantitative findings closely paralleled the redistribution of cell populations observed in the flow cytometric dot plots and indicate that simultaneous exposure to GA and DTX induced the strongest apoptotic response in MDA-MB-231 cells.

### 3.3. TALI Image-Based Cytometry Confirms Enhanced Apoptotic Cell Death Induced by the GA–DTX Combination

**Figure 4:**
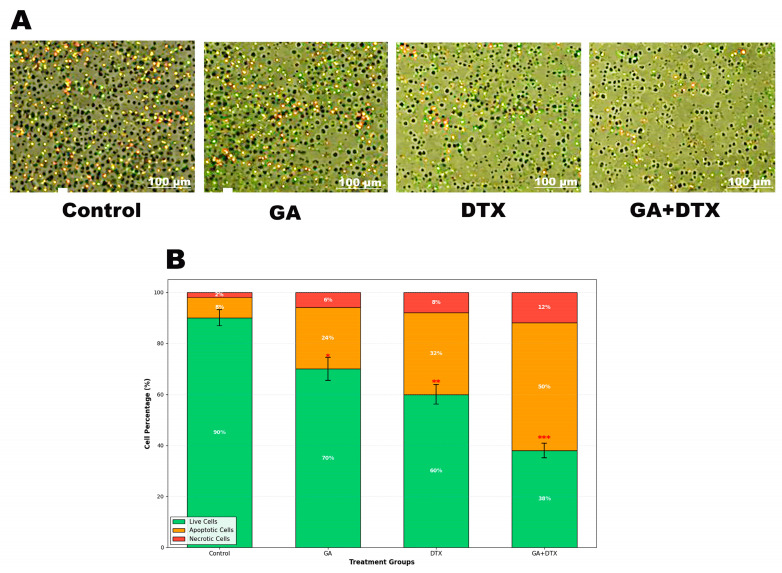
TALI^®^ image-based cytometric analysis of cell viability and cell death in MDA-MB-231 cells after 48 h of treatment with GA, DTX, or the combined regimen. (**A**) Representative fluorescence images acquired with the TALI^®^ Image-Based Cytometer. Viable cells exhibit green fluorescence, whereas apoptotic and membrane-compromised cells are detected by orange-red fluorescence. Images are representative of three independent biological experiments. Scale bar = 100 µm. (**B**) Quantitative evaluation of viable, apoptotic, and necrotic cell populations determined by TALI^®^ image-based cytometry. Cell populations are displayed as viable (green), apoptotic (orange), and necrotic (red). Data are presented as percentages of the total cell population (mean ± SD, *n* = 3). Statistical significance was determined by one-way ANOVA followed by Tukey’s multiple-comparison test (* *p* < 0.05, ** *p* < 0.01, *** *p* < 0.001 versus the untreated control group).

The pro-apoptotic effect identified by flow cytometry was further verified using the TALI^®^ image-based cytometry system after 48 h of treatment with GA, DTX, or their combination (Figure 4A,B). Fluorescence imaging demonstrated a treatment-dependent redistribution of cell populations. Relative to untreated cultures, exposure to either GA or DTX reduced the proportion of viable cells and increased the number of apoptotic cells, whereas the combined treatment produced the most pronounced alteration in cellular status (Figure 4A).

Quantitative analysis supported these observations (Figure 4B). Viable cells represented 90% of the untreated population but declined to 70% following GA treatment (* *p* < 0.05), 60% after DTX treatment (** *p* < 0.01), and 38% in the GA + DTX group (*** *p* < 0.001). In parallel, the apoptotic fraction increased from 8% in untreated cells to 24%, 32%, and 50% after treatment with GA, DTX, and the combined regimen, respectively. Necrosis remained comparatively limited, increasing from 2% in control cultures to 6%, 8%, and 12% in the respective treatment groups (Figure 4B).

Although the numerical values obtained with TALI^®^ image-based cytometry were not identical to those generated by Annexin V-FITC/PI flow cytometry, this difference was expected because the two methods are based on different analytical platforms and classification algorithms. Importantly, both techniques demonstrated the same biological trend, confirming that combined treatment with GA and DTX induced substantially greater apoptosis than either monotherapy in MDA-MB-231 cells.

### 3.4. Combined GA and DTX Treatment Alters β-Tubulin Organization in MDA-MB-231 Cells

**Figure 5:**
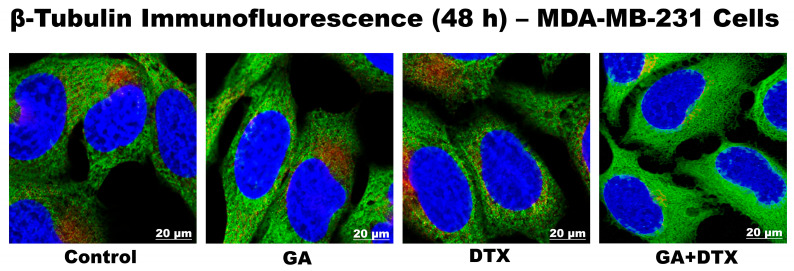
Immunofluorescence analysis of β-tubulin organization in MDA-MB-231 cells following 48 h of treatment with GA, DTX, or the combined regimen. β-Tubulin staining is displayed in green, whereas nuclei are counterstained with DAPI (blue). Control cells exhibited a regular and well-defined microtubule network. Treatment with either GA or DTX altered the β-tubulin staining pattern, while the combined treatment produced the most pronounced disruption of microtubule organization together with evident cell shrinkage and a rounded cellular morphology. Images are representative of three independent biological experiments. Scale bar = 20 µm.

Immunofluorescence staining of β-tubulin was performed to examine treatment-associated changes in microtubule organization after 48 h of drug exposure (Figure 5). Control MDA-MB-231 cells displayed a regular cytoplasmic β-tubulin staining pattern with a continuous and well-organized microtubule network. Exposure to GA produced only limited alterations in β-tubulin distribution and was accompanied by relatively minor changes in cellular morphology.

A more pronounced effect was observed following DTX treatment, where β-tubulin staining appeared condensed and irregular, indicating disruption of the normal microtubule architecture. The combination of GA and DTX produced the most extensive structural alterations. Under this treatment condition, β-tubulin organization was markedly disrupted, and the cells exhibited obvious shrinkage together with a rounded morphology. These morphological features were consistent with the increased apoptotic activity demonstrated in the preceding experiments and further support the enhanced cytotoxic action of the combined treatment in MDA-MB-231 cells (Figure 5). The immunofluorescence images presented in Figure 5 are representative qualitative observations intended to illustrate treatment-associated changes in β-tubulin organization. Quantitative evaluation of apoptosis and treatment efficacy was performed using complementary assays, including flow cytometry, TALI^®^ image-based cytometry, and qRT-PCR, as presented in the subsequent analyses.

### 3.5. Combined GA and DTX Treatment Reduces Pro-Inflammatory Cytokine Secretion

**Figure 6:**
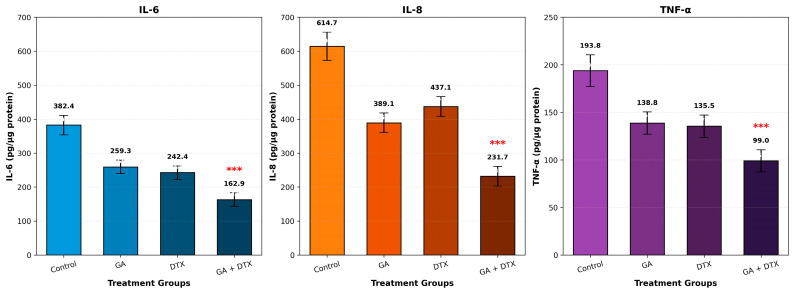
Secretion of IL-6, IL-8, and TNF-α by MDA-MB-231 cells following 48 h of treatment with GA, DTX, or the combined regimen. Cytokine concentrations in conditioned media were determined by ELISA and normalized to total cellular protein content (pg/μg protein). Treatment with either GA or DTX reduced the release of all three cytokines relative to untreated controls, whereas the greatest reduction was observed following combined exposure to both agents. Data are presented as mean ± SD from three independent biological experiments (*n* = 3). Statistical analysis was performed using one-way ANOVA followed by Tukey’s multiple-comparison test (*** *p* < 0.001 versus the untreated control group).

Changes in the inflammatory secretory profile of MDA-MB-231 cells were evaluated by quantifying IL-6, IL-8, and TNF-α levels in conditioned media after 48 h of treatment using ELISA (Figure 6). Compared with untreated cells, exposure to either GA or DTX was associated with a reduction in the secretion of all three cytokines. Untreated cultures released 382.4 ± 28.6 pg/μg protein of IL-6, 614.7 ± 41.3 pg/μg protein of IL-8, and 193.8 ± 16.7 pg/μg protein of TNF-α. Following GA treatment, these values declined to 259.3 ± 17.4, 389.1 ± 26.8, and 138.8 ± 10.6 pg/μg protein, respectively. Similar reductions were observed after DTX exposure, with cytokine concentrations of 242.4 ± 20.3 pg/μg protein for IL-6, 437.1 ± 23.9 pg/μg protein for IL-8, and 135.5 ± 11.8 pg/μg protein for TNF-α.

The combined GA + DTX treatment produced the greatest attenuation of cytokine release. IL-6 levels decreased to 162.9 ± 19.2 pg/μg protein (*** *p* < 0.001), IL-8 secretion was reduced to 231.7 ± 24.5 pg/μg protein (*** *p* < 0.001), and TNF-α levels declined to 99.0 ± 12.4 pg/μg protein (*** *p* < 0.001) relative to untreated controls (Figure 6). These results indicate that simultaneous exposure to GA and DTX suppressed the production of all three pro-inflammatory cytokines more effectively than either monotherapy.

### 3.6. Combined GA and DTX Treatment Inhibits the Migratory Capacity of MDA-MB-231 Cells

**Figure 7:**
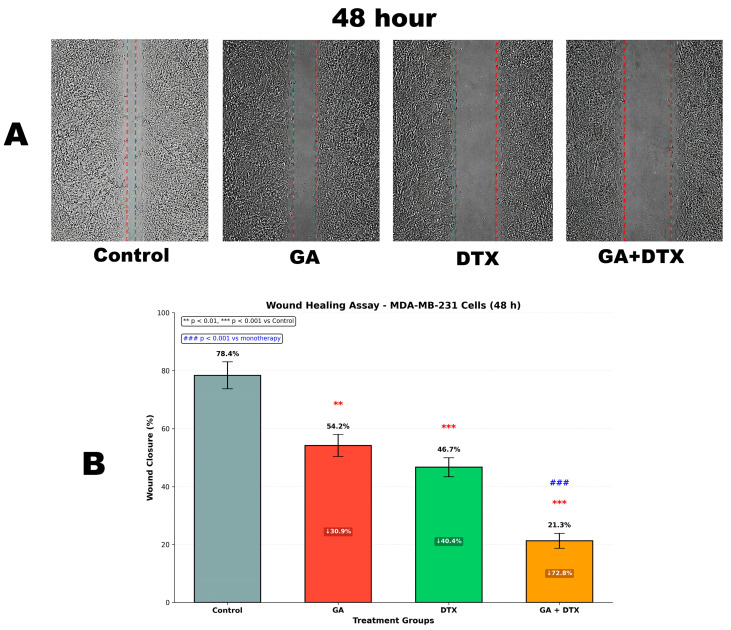
Effect of GA, DTX, and the combined regimen on wound closure by MDA-MB-231 cells after 48 h of treatment. (**A**) Representative phase-contrast images illustrating the remaining wound area in each experimental group. Red dashed lines indicate the wound margins. (**B**) Quantitative analysis of wound closure expressed as the percentage of the initial wound area closed after 48 h. Data are presented as mean ± SD from three independent biological experiments (*n* = 3). Statistical significance was determined using one-way ANOVA followed by Tukey’s multiple-comparison test (** *p* < 0.01 and *** *p* < 0.001 versus the untreated control group; ### *p* < 0.001 versus both monotherapy groups).

The migratory response of MDA-MB-231 cells following exposure to GA, DTX, or the combined treatment was assessed by a wound-healing assay (Figure 7A,B). Untreated cultures exhibited extensive closure of the scratched area after 48 h, reflecting the high migratory capacity of these cells. Both monotherapies slowed wound closure compared with the control group, whereas the combination treatment produced the most evident inhibition, with a substantial portion of the initial wound area remaining unclosed at the end of the experiment (Figure 7A).

Quantitative analysis supported the morphological observations (Figure 7B). Wound closure reached 78.4 ± 4.6% in untreated cells after 48 h. Treatment with GA reduced wound closure to 54.2 ± 3.8% (** *p* < 0.01), while DTX further decreased this value to 46.7 ± 3.3% (*** *p* < 0.001 versus untreated control). The lowest wound closure was measured in the GA + DTX group, where only 21.3 ± 2.6% of the wound area was closed after 48 h (*** *p* < 0.001 versus untreated control; ### *p* < 0.001 versus both monotherapy groups). These results demonstrate that simultaneous treatment with GA and DTX impaired the migratory ability of MDA-MB-231 cells more effectively than either compound alone.

### 3.7. Combined GA and DTX Treatment Modulates the Expression of Apoptosis-, Inflammation-, and Cell Cycle-Related Genes

**Figure 8:**
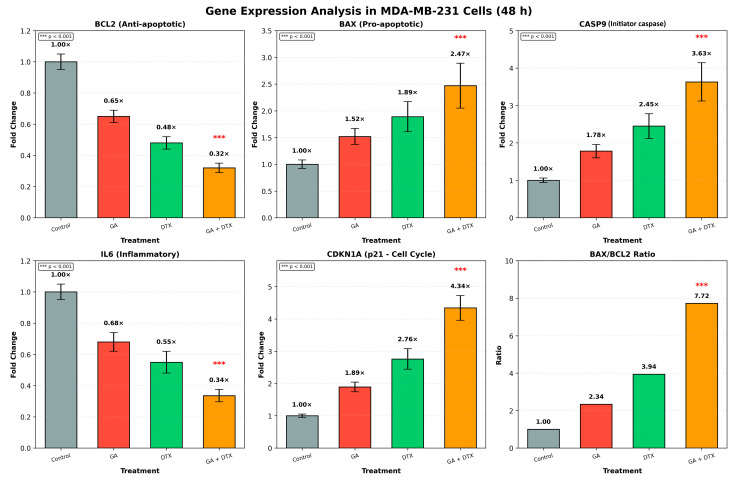
qRT-PCR analysis of treatment-induced changes in gene expression in MDA-MB-231 cells following 48 h of exposure to GA, DTX, or the combined regimen. Relative mRNA expression levels of *BCL2*, *BAX*, *CASP9*, *IL6*, and *CDKN1A* were determined after normalization to the geometric mean of the reference genes *GAPDH* and *ACTB* using the 2^−ΔΔCt^ method. Expression levels are presented as fold changes relative to the untreated control group. The *BAX*/*BCL2* expression ratio was calculated from the normalized data to further assess the balance between pro-apoptotic and anti-apoptotic signaling. Data are presented as mean ± SD from three independent biological experiments (*n* = 3). Statistical significance was determined using one-way ANOVA followed by Tukey’s multiple-comparison test (*** *p* < 0.001 versus the untreated control group).

Transcriptional responses to GA, DTX, and the combined treatment were examined by qRT-PCR after 48 h to determine whether the observed phenotypic alterations were accompanied by changes in the expression of genes involved in apoptosis, inflammation, and cell-cycle regulation (Figure 8). Among the treatment groups, the combination of GA and DTX produced the largest overall effect on gene expression.

Combined treatment markedly reduced the expression of the anti-apoptotic gene *BCL2* to 0.32 ± 0.03-fold relative to untreated cells (*** *p* < 0.001), whereas expression of the pro-apoptotic genes *BAX* and *CASP9* increased to 2.47 ± 0.42-fold and 3.63 ± 0.51-fold, respectively (*** *p* < 0.001). Consequently, the *BAX*/*BCL2* expression ratio increased progressively across the treatment groups and reached its highest value (7.72) in cells exposed to the combined regimen (Figure 8).

A comparable trend was observed for the remaining genes analyzed. Expression of *IL6* decreased significantly to 0.34 ± 0.04-fold (*** *p* < 0.001), while *CDKN1A* expression increased to 4.34 ± 0.38-fold (*** *p* < 0.001) relative to untreated controls. Treatment with either GA or DTX alone produced changes in the same direction, although the magnitude of these responses was consistently lower than that observed following combined treatment (Figure 8).

### 3.8. Bioinformatic Analysis Predicts Key Molecular Targets and Biological Pathways Associated with the GA–DTX Combination

**Figure 9:**
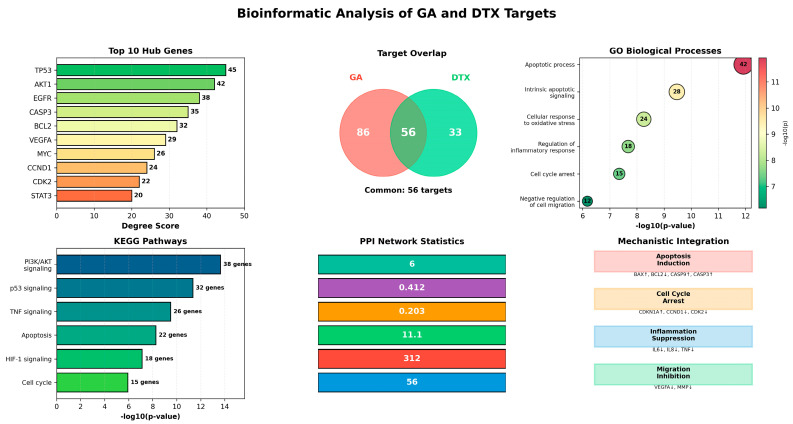
Bioinformatic characterization of the predicted molecular targets and signaling pathways associated with the combined activity of GA and DTX. Analysis of the compound target datasets identified 56 shared targets that were subsequently used for network construction and functional enrichment analyses. The generated PPI network comprised 56 nodes interconnected by 312 edges. GO enrichment analysis demonstrated significant associations with apoptosis, intrinsic apoptotic signaling, cellular responses to oxidative stress, regulation of inflammatory responses, cell-cycle regulation, and regulation of cell migration. KEGG pathway analysis identified the PI3K/AKT, p53, TNF, apoptosis, HIF-1, and cell-cycle pathways as the most significantly enriched signaling pathways. All molecular interactions and enriched pathways shown are based on computational network analyses and should therefore be interpreted as predictive bioinformatic findings rather than experimentally validated molecular events.

To complement the experimental findings, bioinformatic analyses were performed to identify candidate molecular targets and signaling pathways potentially involved in the response to combined GA and DTX treatment. Target prediction identified 142 proteins associated with GA and 89 associated with DTX, of which 56 were common to both compounds and linked to breast cancer-related biological processes (Figure 9). Analysis of these shared targets generated a highly interconnected PPI network comprising 56 nodes and 312 edges. Functional enrichment analysis indicated significant associations with apoptosis, intrinsic apoptotic signaling, cellular responses to oxidative stress, inflammatory regulation, cell-cycle control, and cell migration. KEGG pathway analysis further identified the PI3K/AKT, p53, TNF, apoptosis, HIF-1, and cell-cycle pathways as the most significantly enriched signaling pathways (Figure 9).

**Figure 10:**
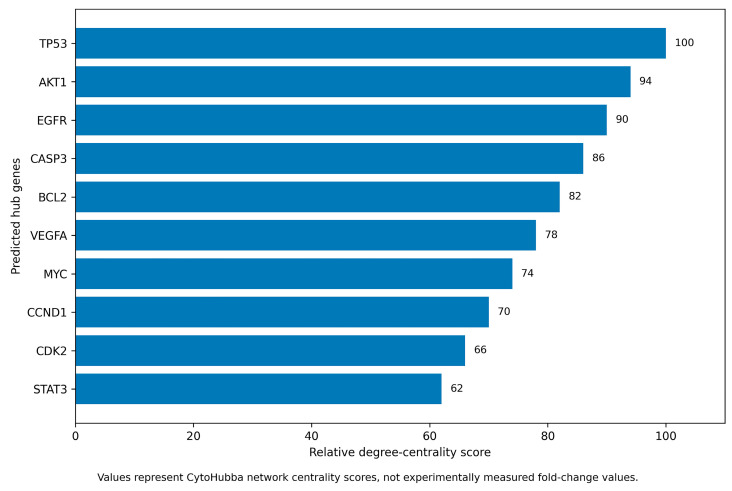
Prioritization of the predicted hub genes within the shared GA–DTX target network based on PPI topology. Hub genes were ranked according to degree-centrality analysis performed with the CytoHubba plugin in Cytoscape. *TP53*, *AKT1*, *EGFR*, *CASP3*, *BCL2*, *VEGFA*, *MYC*, *CCND1*, *CDK2*, and *STAT3* showed the highest degree-centrality scores, indicating that these genes occupy the most highly connected positions within the predicted interaction network. Higher degree-centrality values reflect greater network connectivity and increased predicted topological relevance. Because these rankings were generated exclusively through computational network analysis, they should not be interpreted as experimentally determined gene-expression levels or fold-change measurements. Instead, the identified hub genes represent candidate molecular targets that warrant further experimental validation.

Network topology analysis using the CytoHubba plugin was subsequently performed to prioritize the most influential nodes within the shared target network. *TP53*, *AKT1*, *EGFR*, *CASP3*, *BCL2*, *VEGFA*, *MYC*, *CCND1*, *CDK2*, and *STAT3* were identified as the highest-ranked hub genes according to degree-centrality analysis (Figure 10). Because these results originate from computational network-based predictions, they should be interpreted as candidate molecular associations rather than experimentally validated changes in gene expression. Nevertheless, the enrichment of pathways associated with apoptosis, inflammatory signaling, and cell-cycle regulation was consistent with the biological responses observed in the experimental analyses performed in MDA-MB-231 cells.

## 4. Discussion

The present study systematically investigated the biological effects of combining GA with DTX in the MDA-MB-231 TNBC model through an integrated experimental strategy that included cytotoxicity assessment, drug-interaction analysis, apoptosis assays, inflammatory cytokine measurements, wound-healing analysis, β-tubulin IF, gene-expression profiling, and bioinformatic network analyses. HaCaT human keratinocytes were examined in parallel as a non-malignant epithelial comparator to obtain a preliminary indication of differential cellular sensitivity. Across these complementary experimental approaches, GA consistently potentiated the anticancer activity of DTX in MDA-MB-231 cells. This enhanced response was reflected by stronger induction of apoptosis, disruption of β-tubulin organization, attenuation of pro-inflammatory cytokine secretion, impaired wound closure, modulation of apoptosis- and cell cycle-associated genes, and bioinformatic predictions that closely paralleled the experimental observations. Collectively, these findings indicate that GA may strengthen the antitumor efficacy of DTX through multiple interconnected biological mechanisms and provide a rationale for further mechanistic studies of this combination [25,26,27].

One of the most notable findings of the present work was the synergistic interaction between GA and DTX, demonstrated by CI values consistently below unity. This observation is clinically relevant because the therapeutic use of DTX continues to be constrained by adverse effects such as myelosuppression, peripheral neuropathy, and fluid retention [6]. The ability of GA to potentiate DTX-mediated cytotoxicity raises the possibility that dose-optimization strategies may be explored in future preclinical studies, although this hypothesis requires validation in advanced experimental models before any conclusions regarding treatment-associated toxicity can be drawn. Similar chemosensitizing properties of GA have previously been described in combination with cisplatin, doxorubicin, and paclitaxel in several experimental cancer models, although the molecular basis of these interactions appears to vary depending on the cellular context [25,26,27].

The enhanced activity observed with the combined treatment is likely attributable to the complementary mechanisms through which GA and DTX influence cancer-cell survival. DTX interferes with microtubule dynamics by promoting microtubule stabilization, thereby inducing G2/M arrest and triggering apoptotic signaling through phosphorylation and functional inactivation of BCL-2 together with caspase activation [28]. By comparison, GA has been reported to activate apoptosis through several distinct mechanisms, including mitochondrial membrane depolarization, alteration of the *BCL2*/*BAX* balance, increased ROS generation, suppression of NF-κB and PI3K/AKT signaling, and activation of the JNK pathway [29,30].

These complementary mechanisms appear to converge on the intrinsic mitochondrial apoptotic pathway, providing a plausible explanation for the enhanced apoptotic response detected after combined treatment. In agreement with this interpretation, combination-treated cells displayed increased caspase-9 immunoreactivity, a higher proportion of apoptotic cells, and marked transcriptional changes in apoptosis-related genes. Specifically, expression of *BAX* and *CASP9* increased, whereas *BCL2* expression declined, resulting in a substantial elevation of the *BAX*/*BCL2* ratio. Because *CASP9* functions as the initiator caspase of the intrinsic mitochondrial apoptotic pathway, its increased expression supports activation of upstream apoptotic signaling. Likewise, the simultaneous decrease in *BCL2* and increase in *BAX* indicate a shift toward a pro-apoptotic cellular phenotype. Since BCL-2 has been implicated in the development of chemoresistance in breast cancer, coordinated regulation of BCL-2 family members by GA and DTX may represent one mechanism contributing to the stronger apoptotic response observed following combination treatment [31,32,33]. Although prolonged G2/M arrest may contribute to apoptosis induction by promoting mitotic stress, the present study does not establish a direct causal relationship between cell-cycle arrest and apoptotic cell death. Instead, the enhanced apoptotic response observed following combination treatment is more likely to reflect the coordinated activation of multiple biological processes, including mitochondrial apoptotic signaling, alterations in *BCL2* family gene expression, caspase-9 activation, inflammatory cytokine modulation, and cytoskeletal remodeling. Therefore, apoptosis should be interpreted as the result of multiple parallel mechanisms rather than as an exclusive consequence of G2/M arrest.

Immunofluorescence evaluation of β-tubulin demonstrated pronounced treatment-associated remodeling of the microtubule network, particularly in DTX- and GA + DTX-treated cells. These structural alterations are compatible with the established mechanism of action of DTX and are consistent with the accompanying cell-cycle disturbances. Nevertheless, the present findings do not establish a direct mechanistic link between β-tubulin reorganization and apoptosis induction. Consequently, the IF results should be interpreted as morphological evidence supporting cytoskeletal remodeling during treatment rather than definitive proof that microtubule disruption directly caused apoptotic cell death [34]. Moreover, the present study was not designed to determine whether GA directly modulates microtubule dynamics or enhances the interaction of DTX with β-tubulin. Therefore, the enhanced β-tubulin reorganization observed following combination treatment should be interpreted as evidence of treatment-associated cytoskeletal remodeling rather than proof of a direct molecular interaction between GA and β-tubulin. Clarification of the precise molecular mechanism will require dedicated biochemical, biophysical, and structural studies in future investigations.

The decrease in IL-6, IL-8, and TNF-α secretion detected after treatment may also contribute to the overall anticancer effects observed in MDA-MB-231 cells. These cytokines participate in signaling pathways that promote tumor-cell proliferation, survival, angiogenesis, migration, and therapeutic resistance in TNBC and other aggressive malignancies [35,36]. Compared with either monotherapy, the combined GA + DTX treatment produced a greater reduction in all three cytokines, suggesting that suppression of inflammatory mediator production accompanies its antiproliferative and pro-apoptotic activities. However, because cytokine quantification was performed exclusively in conditioned media obtained from tumor-cell monocultures, these findings reflect altered cytokine secretion by cancer cells rather than direct evidence of tumor microenvironment remodeling or immune regulation. Validation in co-culture systems incorporating stromal and immune cells, as well as in vivo studies, will be necessary to clarify the biological significance of these observations within the tumor microenvironment [37].

A similar consideration applies to the wound-healing assay. Although the combination treatment produced the greatest inhibition of wound closure, the experimental design does not permit discrimination between reduced cell migration, diminished proliferation, and treatment-induced cell death, all of which may contribute to delayed wound closure. Because proliferation inhibitors such as mitomycin-C were not incorporated into the assay, the results should be interpreted as evidence of impaired wound closure rather than definitive confirmation of a direct anti-migratory effect. Nevertheless, these findings are compatible with previous reports indicating that DTX affects cytoskeletal dynamics, whereas GA has been associated with modulation of migration-related signaling pathways, including matrix metalloproteinases and focal adhesion kinase signaling [38,39,40]. Additional studies specifically designed to distinguish migratory effects from antiproliferative responses will be required.

The bioinformatic analyses complemented the experimental findings by identifying molecular pathways that may participate in the biological response to combined GA and DTX treatment. Network analysis highlighted *TP53*, *AKT1*, *EGFR*, *CASP3*, *BCL2*, and *STAT3* as major hub genes and demonstrated enrichment of the PI3K/AKT, p53, apoptosis, and cell-cycle pathways. These computational predictions are consistent with the experimentally observed induction of apoptosis, altered expression of *BCL2*, *BAX*, *CASP9*, and *CDKN1A*, and suppression of inflammatory mediator production. Nevertheless, the hub genes identified by network analysis were not experimentally validated in the present study and should therefore be regarded as computationally predicted targets rather than confirmed molecular mediators. Accordingly, these findings primarily generate hypotheses that warrant future experimental validation focusing on PI3K/AKT, p53, and related signaling pathways [41,42,43]. Future studies incorporating complementary in silico approaches, such as molecular docking and molecular dynamics simulations, may further improve mechanistic understanding of the potential interactions between GA and key regulatory proteins identified in the present study.

HaCaT keratinocytes were included as a widely accepted spontaneously immortalized, non-tumorigenic human epithelial cell model for preliminary cytotoxicity assessment under identical experimental conditions [44,45]. Originally established from adult human skin, HaCaT cells retain stable differentiation characteristics while remaining non-tumorigenic, making them one of the most extensively used non-malignant epithelial cell models in in vitro toxicological and pharmacological studies [44]. More recently, HaCaT cells have continued to serve as a well-established experimental model for investigating epidermal homeostasis and epithelial cell biology, further supporting their utility in mechanistic in vitro studies [45]. Nevertheless, HaCaT cells originate from epidermal keratinocytes rather than normal mammary epithelial tissue. Therefore, the differential cytotoxic responses observed between MDA-MB-231 and HaCaT cells should not be interpreted as evidence of breast tissue-specific selectivity. Instead, the present comparison provides a preliminary indication of the differential sensitivity of malignant and non-malignant epithelial cells under identical experimental conditions. Comparison of the cytotoxic responses of MDA-MB-231 and HaCaT cells demonstrated greater sensitivity of the malignant cells to GA, as indicated by the lower IC_50_ value obtained in MDA-MB-231 cells. In contrast, DTX exhibited comparable IC_50_ values in both cell lines, suggesting relatively limited selectivity under the present experimental conditions. It should also be emphasized that all mechanistic analyses, including apoptosis, β-tubulin IF, cytokine secretion, wound healing, and gene-expression profiling, were performed exclusively in MDA-MB-231 cells. Consequently, the mechanistic conclusions presented here should be considered specific to this TNBC model and should not be generalized to HaCaT cells without additional experimental evidence. Future studies employing non-tumorigenic mammary epithelial models, such as MCF-10A, together with additional TNBC cell lines, will be important to further validate the tissue-specific differential sensitivity observed in the present study.

Several limitations should be considered when interpreting the present findings. First, the study relied on a single TNBC cell line, which does not fully represent the biological heterogeneity of this breast cancer subtype. Although HaCaT keratinocytes are widely used as a non-malignant epithelial cell model for preliminary cytotoxicity assessment, they do not represent normal mammary epithelial tissue. Therefore, the differential cytotoxic responses observed between MDA-MB-231 and HaCaT cells should not be interpreted as evidence of breast tissue-specific selectivity. Instead, the present comparison provides an initial indication of the differential sensitivity between malignant and non-malignant epithelial cells under identical experimental conditions. Future studies should validate these findings using non-tumorigenic mammary epithelial models, such as MCF-10A, to better define the tissue-specific therapeutic window of the GA + DTX combination. Third, the proposed molecular mechanisms were supported primarily by IF and mRNA analyses, whereas comprehensive protein-level validation by Western blotting was not performed for most signaling molecules. Fourth, the wound-healing assay did not include proliferation-blocking agents such as mitomycin-C, precluding definitive separation of migration-dependent and proliferation-dependent effects. Fifth, the bioinformatic analyses represent computational predictions generated through target-network enrichment and should be interpreted accordingly. Finally, all experiments were conducted under conventional two-dimensional culture conditions. The absence of three-dimensional tumor spheroid models and in vivo validation limits direct translation of these findings to clinical settings. In addition, the relatively poor bioavailability of GA represents an important challenge for its clinical translation despite its broad pharmacological and anticancer potential [46,47,48,49]. Pharmacokinetic studies have demonstrated that GA is rapidly absorbed after oral administration but also undergoes rapid metabolism and elimination, resulting in limited systemic bioavailability [47,48]. In particular, extensive phase II biotransformation, including glucuronidation, sulfation, and methylation, substantially reduces the amount of free GA available for therapeutic activity in vivo [47]. Consequently, the intratumoral concentrations required to reproduce the synergistic effects observed under controlled in vitro conditions may be difficult to achieve using conventional formulations. To overcome these pharmacokinetic limitations, several advanced nano-delivery systems, including polymeric nanoparticles, dendrimers, and nanodots, have been developed to improve the stability, bioavailability, and tissue delivery of GA [47]. Nevertheless, despite these promising advances, further pharmacokinetic investigations, optimized formulation strategies, and rigorous preclinical and clinical validation remain essential before the therapeutic potential of the GA–DTX combination can be fully translated into clinical practice [49]. Future investigations should integrate three-dimensional tumor spheroid models and well-designed in vivo studies to determine whether the synergistic antitumor effects observed in the present in vitro model can be reproduced under physiologically relevant conditions. In particular, preclinical animal studies should evaluate antitumor efficacy, systemic toxicity, pharmacokinetic behavior, and intratumoral drug distribution of the GA–DTX combination, together with protein-level validation and functional pathway analyses, to better define its biological activity, safety profile, and potential for future preclinical development.

## 5. Conclusions

The findings of the present study demonstrate that the combination of GA and DTX exerts greater anticancer activity against MDA-MB-231 TNBC cells than either agent administered individually. Synergistic interaction between the two compounds was accompanied by enhanced induction of apoptosis, as independently confirmed by both flow cytometry and TALI^®^ image-based cytometry, together with pronounced alterations in β-tubulin organization, suppression of pro-inflammatory cytokine secretion, impaired wound closure, and coordinated regulation of apoptosis- and cell cycle-related gene expression. In addition, the bioinformatic analyses predicted the involvement of apoptosis-, cell cycle-, and inflammation-related signaling pathways, and these computational findings were generally consistent with the experimental observations, although they require further biological validation.

Comparison of the cytotoxic responses of malignant and non-malignant cells demonstrated that MDA-MB-231 cells were more susceptible to GA than HaCaT keratinocytes, whereas DTX exhibited similar cytotoxic activity in both cell lines under the experimental conditions used in this study. Overall, these findings support the potential role of GA as a candidate chemosensitizing agent capable of enhancing the biological activity of DTX in this in vitro TNBC model. However, because the present investigation was restricted to in vitro experiments and computational network analyses, these findings should be considered proof-of-concept evidence. Additional studies involving multiple breast cancer models, three-dimensional culture systems, pharmacokinetic evaluation, protein-level mechanistic validation, and well-designed in vivo preclinical models will be required before the biological effects observed in the present study can be translated into potential clinical applications.

## Figures and Tables

**Table 1:** Primer sequences used for qRT-PCR analysis.

Gene	Forward Primer (5′→3′)	Reverse Primer (5′→3′)
*BCL-2*	ATGCCTTTGTGGAACTATATGGC	GGTATGCACCCAGAGTGATGC
*BAX*	CCCGAGAGGTCTTTTTCCGAG	CCAGCCCATGATGGTTCTGAT
*CASP9*	GAAGCGAATCAATGGACTCGG	CTTGCACTCCTGCATCAGCTT
*IL6*	AGACAGCCACTCACCTCTTCAG	TTCTGCCAGTGCCTCTTTGCTG
*CDKN1A*	AGGTGGACCTGGAGACTCTCAG	TCCTCTTGGAGAAGATCAGCCG
*GAPDH*	GTCTCCTCTGACTTCAACAGCG	ACCACCCTGTTGCTGTAGCCAA

## Data Availability

The original contributions presented in this study are included in the article. Further inquiries can be directed at the corresponding authors.

## Abbreviations

BP	Biological process
CC	Cellular component
CI	Combination index
DMSO	Dimethyl sulfoxide
DTX	Docetaxel
Fa	Fractional effect
FDR	False discovery rate
GA	Gallic acid
GO	Gene Ontology
IC_50_	Half-maximal inhibitory concentration
IF	Immunofluorescence
KEGG	Kyoto Encyclopedia of Genes and Genomes
MF	Molecular function
PBS	Phosphate-buffered saline
PI	Propidium iodide
PPI	Protein–protein interaction
ROS	Reactive oxygen species
TNBC	Triple-negative breast cancer
